# Higher central arterial wall viscosity and stiffness in resistance‐trained young men

**DOI:** 10.14814/phy2.70526

**Published:** 2025-09-02

**Authors:** Hiroshi Kawano, Nobuhiro Nakamura, Kumpei Tanisawa, Masayuki Konishi, Masashi Miyashita, Kenta Yamamoto, Shizuo Sakamoto, Motohiko Miyachi

**Affiliations:** ^1^ Faculty of Letters Kokushikan University Setagaya Tokyo Japan; ^2^ Faculty of Sport Sciences Waseda University Tokorozawa Saitama Japan; ^3^ Faculty of Health Promotional Sciences Tokoha University Hamamatsu Shizuoka Japan; ^4^ School of Sport, Exercise and Health Sciences Loughborough University Loughborough Leicestershire UK; ^5^ Department of Sports Science and Physical Education The Chinese University of Hong Kong Shatin New Territories Hong Kong; ^6^ Faculty of Pharmaceutical Sciences Teikyo Heisei University Nakano Tokyo Japan

**Keywords:** arterial compliance, arterial stiffness, arterial wall viscosity, cardiac function, resistance training

## Abstract

Resistance training reduces arterial compliance. Although cardiac pulsatile energy is distributed between arterial compliance and arterial wall viscosity, arterial wall viscosity in resistance‐trained men remains unclear. Therefore, this study aimed to examine whether resistance‐trained men exhibit increased arterial wall viscosity. This cross‐sectional study included 28 young men (18–25 years), with 12 resistance‐trained men and 16 non‐trained sedentary controls. We measured arterial wall viscosity, arterial compliance, carotid and brachial blood pressures, peak oxygen uptake, and left ventricular mass and functions. Resistance‐trained men had higher arterial wall viscosity (3064 ± 1208 vs. 1993 ± 831 mmHg·s/mm: *p* = 0.010) and lower compliance (0.093 ± 0.029 vs. 0.153 ± 0.054 mm^2^/mmHg: *p* = 0.003) than controls, with an elevated beta‐stiffness index (7.90 ± 2.80 vs. 5.86 ± 1.26 AU: *p* = 0.015). Intima‐media thickness and brachial systolic and diastolic blood pressures, peak oxygen uptake, and stroke volume did not differ between groups, although there were significantly higher carotid systolic blood pressure and pulse pressure in resistance‐trained men compared to controls. Resistance‐trained men showed concentric left ventricular hypertrophy. The present study indicated that central arterial wall viscosity is higher and arterial compliance is lower in resistance‐trained men.

## 1 INTRODUCTION

Central arterial elasticity (compliance) is known to be reduced by resistance training (Miyachi et al., 2003; Miyachi et al., 2004), and this finding has been confirmed by a meta‐analysis (Miyachi, 2012). The central elastic arteries function as viscoelastic conduits, serving both as pathways for blood flow and as buffers to smooth the pulsatile energy generated by cardiac contractions. Arterial wall viscosity serves as a mechanism for dissipating cardiac pulsatile energy that would otherwise be stored as elastic energy (Bertram, 1980; Bodley, 1976; Nichols et al., 1998; Taylor, 1966). Although central elastic arteries possess viscoelastic properties, no study has investigated how arterial wall viscosity adapts in response to resistance training, despite previous reports indicating that arterial compliance decreases with resistance training (Miyachi et al., 2003, 2004). Given that central arteries are inherently viscoelastic structures, it is important to evaluate not only their elastic properties but also their viscous properties as critical components of arterial function in response to resistance training.

Endurance training induces enhanced left ventricular mass and stroke volume (Scharhag et al., 2002). Endurance training is associated with higher arterial compliance in middle‐aged and elderly men, but this effect is not observed in young men (Tanaka et al., 2000). We reported that endurance‐trained young men have greater arterial wall viscosity and not different arterial compliance as compared with age‐matched control men (Kawano et al., 2021). These findings suggest that endurance‐trained men dissipate greater pulsatile energy from the heart as a viscosity during the conversion of cardiac pulsatile energy into arterial elastic energy because of reaching the upper limit dynamic arterial compliance in endurance‐trained young men. On the other hand, resistance training induces concentric hypertrophy in the left ventricle characterized by no change in stroke volume and a reduction in arterial compliance (Miyachi et al., 2004). Considering the coupling adaptations of the left ventricle and arterial viscoelasticity response to endurance training, we can hypothesize that resistance‐trained men exhibit increased arterial wall viscosity and reduced arterial compliance. To elucidate this hypothesis, we designed a cross‐sectional study to compare arterial wall viscosity and compliance as well as left ventricular indices between resistance‐trained young athletes and age‐matched sedentary control men.

## 2 METHODS

### 2.1 Subjects and ethical approval

A total of 28 healthy young men aged 18–25 years participated in the present study (Table 1). Sixteen control men were recruited through various forms of advertisement and had not engaged in an endurance and/or resistance training program. Twelve resistance‐trained men (shot put, discus, and javelin throwing athletes) were recruited, who had been resistance training for at least 4 years, 4–6 times a week, except for the throwing exercises. All subjects who were taking medications, had ever used anabolic steroids, or who had significant carotid intima–media thickening (<1.1 mm), plaque formation, hypertension (>140/90 mmHg), and/or other characteristics of atherosclerosis [ankle‐brachial index (ABI) < 0.9] were excluded from the study. The purpose, procedures, and risks of the study were explained to each subject, all of whom provided written informed consent before participating in the study, which was approved by the Human Research Ethical Committee of Kokushikan University (Approval NO. R1‐05) and the Human Research Ethical Committee of Waseda University (Approval NO. 2011‐203). This study was carried out in accordance with the guidelines of the Declaration of Helsinki.

**TABLE 1:** Subject characteristics.

	Control	Resistance‐trained	*p* Value
*N*	16	12	
Age, years	20.5 ± 1.6	20.0 ± 1.7	0.432
Height, cm	170.0 ± 5.5	176.7 ± 6.1	**0.005**
Body weight, kg	68.9 ± 7.7	90.3 ± 11.0	**<0.001**
Body mass index, kg/m^2^	23.9 ± 2.6	28.9 ± 3.4	**<0.001**
% Body fat, %	18.0 ± 5.8	22.0 ± 4.1	0.056
Peak heart rate, bpm	192.3 ± 11.5	185.0 ± 8.8	0.080
VO_2peak_, mL/min	2605 ± 490	3147 ± 261	**0.002**
VO_2peak_/body weight, mL/kg/min	37.8 ± 5.0	35.2 ± 4.4	0.164

*Note*: Data are means ± SD. *p* values indicating statistical significance (*p* < 0.05) are shown in bold.

Abbreviations: *N*, no. of subjects; VO_2_peak, peak oxygen consumption.

The subjects abstained from caffeine and fasted for at least 4 h (12 h overnight fast was used to determine cardiovascular functions) before they were tested. Resistance‐trained men were studied 24 h after their last exercise training session to minimize the acute effects of exercise. All measurements were carried out under comfortable laboratory conditions between 09:00 am and 12:00 am. The aerobic capacity assessment was performed after the other tests.

### 2.2 Carotid arterial diameter waveform

We measured the carotid arterial diameter waveform using a B‐mode ultrasound device (LOGIQ e; GE Medical Systems, Milwaukee, WI) equipped with a 12.0‐MHz probe. This was done by capturing a longitudinal image of the cephalic portion of the right common carotid artery, about 1–2 cm up from the carotid bulb while the subject was in a supine position. We obtained image sequences from at least 40 cardiac cycles to determine the instantaneous carotid arterial diameter waveform. The electrocardiogram (ECG) was synchronized with these images to average out the carotid diameter waveforms and to calculate the hysteresis loop diameter–pressure in the carotid artery. The images, captured at a frequency of 66 Hz, were transferred to a personal computer, digitized at 636 × 434 pixels with 256 gray levels, and analyzed offline using the auto‐tracking software MoveTr2D (Library Corporation, Tokyo, Japan), as previously described in Kawano et al. (2013).

### 2.3 Carotid arterial pressure waveform

The pressure wave of the carotid artery was recorded at the identical site as the diameter wave, but subsequent to echographic measurement. This was accomplished using a pencil‐type probe equipped with a high‐fidelity strain‐gauge transducer (SPT‐301; Millar Instruments, Houston, TX) (Kelly et al., 1989; Tanaka et al., 2000). To ensure statistical reliability, an average of at least 40 cardiac cycles was obtained, and to assess the hysteresis loop of diameter–pressure within the carotid artery, both the carotid arterial pressure waveform and electrocardiogram (ECG) were simultaneously recorded while subjects were in a supine position. The carotid arterial pressure and ECG waveforms were digitized at a sampling rate of 2000 Hz by connecting each device to a personal computer via an analog‐to‐digital converter (PowerLab; AD Instruments, Bella Vista, NSW, Australia). Given that baseline levels of carotid blood pressure are influenced by the applied hold‐down force, the pressure signal acquired through tonometry was calibrated by aligning the mean and diastolic pressures of the carotid artery with those of the brachial artery (Armentano et al., 1995).

### 2.4 Arterial blood pressure at rest

Chronic resting arterial blood pressure was assessed using a semiautomated devices (Form PWV/ABI; OMRON COLIN Co., Ltd., Tokyo, Japan and HBP‐8000; FUKUDA COLIN Co., Ltd., Tokyo, Japan) across both the brachial and anterior tibial arteries. Measurements were conducted in triplicate with subjects in a supine position as described previously (Kawano et al., 2008).

### 2.5 Peak oxygen uptake

Peak oxygen consumption (VO_2peak_) was quantified during incremental cycle ergometer exercise, starting at 60 watts and increasing by 15 watts per minute, serving as an index of cardiorespiratory fitness. Throughout the exercise protocol, oxygen consumption, heart rate, and ratings of perceived exertion were continuously monitored (Aoyama et al., 2011). Expired gas from the subjects was collected, and the rates of oxygen consumption and carbon dioxide production were measured and averaged over 30‐s intervals using an automated gas analysis system (AE‐310S, Minato Medical Science, Tokyo, Japan). Achievement of VO_2peak_ was confirmed if at least three of the following five criteria were met: VO_2peak_ curve showed a leveling off; the subject's peak heart rate was >90% the age‐predicted maximal heart rate (220—age); the respiratory exchange ratio was >1.1; the subject achieved ratings of perceived exertion of 18; and a pedal speed maintaining below 60 revolutions per minute.

### 2.6 Percent body fat

Body composition was assessed using the bioelectrical impedance analysis method, which demonstrated a day‐to‐day coefficient of variation of 4 ± 2% (Bolanowski & Nilsson, 2001).

### 2.7 Pressure‐dependent analysis of diameter, compliance, and stiffness

As previously mentioned, the pressure‐diameter hysteresis loop was determined using a computerized method, which relied on an ECG trigger for synchronization (Armentano et al., 1991; Barra et al., 1993). The purely elastic pressure‐diameter relationship was calculated using a specialized system named Kaiseki KENTA‐KUN (Kawano et al., 2013). Each cardiac cycle from the recorded pressure and diameter signals, totaling at least 40 beats, was resampled to 256 samples per beat. Subsequently, the average pressure and diameter for each beat were computed. The pressure‐diameter hysteresis loop was then constructed through x‐y plotting of the pressure and diameter waveforms. To derive the purely elastic pressure‐diameter relationship, real pressure (*P*_real_) was converted into elastic pressure (*P*_elastic_) using a first‐order differential equation (Bauer et al., 1979), which accounts for the viscoelastic properties of the arterial wall.

(1) $$P_{\text{elastic}}=P_{\text{real}}-\eta \cdot \frac{\mathrm{dD}}{\mathrm{dt}}$$

In this context, η represents the viscosity index, and dD/dt is the first derivative of the diameter with respect to time. The value of η was incrementally adjusted to decrease the area of the hysteresis loop. This adjustment continued until the loop's area reached its smallest possible size while still maintaining a clockwise direction.

The purely elastic relationship derived from the hysteresis elimination process was modeled using a logarithmic function previously utilized to characterize the elastic properties of large arteries. This model was converted into a diameter‐pressure curve based on a formula that defines the diameter (*D*) as a function of elastic pressure (P_elastic_) incorporating two constants, α and β, established through the fitting process. This methodology aligns with the protocols described in prior studies by Armentano et al. (1995); Armentano et al. (1998).

(2) $$D=\alpha +\beta \cdot \ln\;P_{\text{elastic}}$$

The compliance‐pressure curve was calculated by deriving Equation 2 with respect to pressure (dD/dP_elastic_). The stiffness‐pressure curve was calculated by deriving Equation 2 with respect to pressure (*P*_elastic_/dD). The diameter‐pressure, compliance‐pressure, and stiffness‐pressure curves were all plotted across the same blood pressure range, from 50 to 150 mmHg.

### 2.8 Elastic parameters, viscosity index, dynamic compliance, and beta‐stiffness index

The compliance‐pressure and stiffness‐pressure curves facilitated the calculation of effective compliance and stiffness values at the prevailing mean arterial pressure for each subject. Additionally, these curves enabled the determination of isobaric compliance and stiffness values standardized across all subjects at a uniform pressure level. This standard pressure was chosen arbitrarily as the arithmetic average of mean arterial pressure of all subjects (83.4 mmHg).

The wall viscosity index was calculated using Equation 1 by iteratively adjusting the *η* coefficient upwards until the hysteresis loop area was minimized. The optimal *η* value, which corresponded to the minimal area of the hysteresis loop, was then designated as the viscosity index.

Dynamic arterial compliance and the beta‐stiffness index were evaluated as measures of arterial function, based on established methods from the literature (Green et al., 2004; Kawano et al., 2006, 2008; Miyachi et al., 2003; Parati & Bernardi, 2006; Tanaka et al., 2000). The beta‐stiffness index serves as a measure of dynamic arterial compliance, adjusted for the effects of distending pressure (Hirai et al., 1989). Calculations for dynamic arterial compliance, the beta‐stiffness index, and distensibility were conducted using the following equations:

$$\text{Dynamic arterial compliance}=\frac{\left(D_{1}-D_{0}\right)/D_{0}}{2\cdot \left(P_{1}-P_{0}\right)}\cdot \pi \cdot {D_{0}}^{2}$$

and

$$\text{Beta}-\text{stiffness index}=\;\frac{\ln\left(P_{1}/P_{0}\right)}{\left(D_{1}-D_{0}\right)/D_{0}}$$

and

$$\text{Distensibility}=\;\frac{\left(D_{1}-D_{0}\right)/D_{0}}{P_{1}-P_{0}}$$

### 2.9 Intima‐media thickness

Carotid intima‐media thickness was measured from images obtained using a B‐mode ultrasound device (LOGIQ e; GE Medical Systems, Milwaukee, WI). The ultrasound images were analyzed using image analysis software (ImageJ 1.42; NIH, Bethesda, MD). At least 10 intima‐media thickness measurements were taken at each segment, and the mean values were used for analysis, as described previously (Kawano et al., 2006).

### 2.10 Left ventricular mass and functions

Echocardiography was used to assess left ventricular dimensions, wall thickness, and stroke volume, following established guidelines (Cheitlin et al., 1997). Left ventricular mass was determined using the formula developed by Devereux et al. (1986). Both left ventricular mass and stroke volume were normalized to body surface area. Left ventricular relative wall thickness was calculated as twice the ratio of posterior wall thickness to the left ventricular end‐diastolic diameter.

### 2.11 Statistical analyses

Statistical analyses were conducted using StatView software (SAS Institute, Cary, NC). Continuous variables were compared using unpaired *T* tests. Relations of interest (intima‐media thickness and wall viscosity) were identified by univariate correlational analysis. All data are presented as means ± standard deviation (SD). A *p* value of less than 0.05 was considered statistically significant for all comparisons.

## 3 RESULTS

Table 1 shows the characteristics of control and resistance‐trained men. Height, body weight, and body mass index were higher in resistance‐trained men compared to control men, but there were no significant differences in age or percentage of body fat between the two groups. There was also no significant difference in peak heart rate between the two groups. The absolute value of peak oxygen consumption was significantly higher in resistance‐trained men than in control men; but this difference disappeared when adjusted for body weight.

Table 2 shows vascular indices. Resting heart rate, brachial systolic and diastolic blood pressures, and pulse pressure did not differ significantly between the two groups; however, the mean arterial pressure was higher in resistance‐trained men than in control men. However, carotid systolic blood pressure and pulse pressure were significantly higher in resistance‐trained men compared to control men. Regarding the morphology and distensibility of the carotid artery, there were no significant differences between the two groups in carotid systolic diameter, diastolic diameter, or intima‐media thickness. When intima‐media thickness was adjusted for carotid diastolic diameter (i.e., wall‐to‐lumen ratio), no significant difference was found either; however, the *p* value was 0.0668, suggesting a modest trend toward a higher ratio in the resistance‐trained group.

**TABLE 2:** Vascular indices.

	Control	Resistance‐trained	*p* Value
Resting heart rate, bpm	59.8 ± 7.7	54.6 ± 7.6	0.092
Brachial SBP, mmHg	111.8 ± 8.0	117.3 ± 9.1	0.099
Brachial DBP, mmHg	60.1 ± 7.5	62.0 ± 5.2	0.476
Brachial MAP, mmHg	79.6 ± 7.7	88.5 ± 8.3	**0.007**
Brachial PP, mmHg	51.6 ± 4.1	55.4 ± 5.6	0.054
Carotid SBP, mmHg	110.7 ± 13.9	128.1 ± 14.0	**0.003**
Carotid PP, mmHg	50.6 ± 15.4	66.1 ± 10.3	**0.006**
Carotid systolic diameter, mm	7.09 ± 0.75	6.70 ± 0.69	0.170
Carotid diastolic diameter, mm	6.43 ± 0.69	6.10 ± 0.68	0.209
Intima‐media thickness, mm	0.47 ± 0.06	0.51 ± 0.06	0.118
Wall‐to‐lumen ratio, %	0.074 ± 0.014	0.084 ± 0.013	0.067

*Note*: Data are means ± SD. *p* values indicating statistical significance (*p* < 0.05) are shown in bold.

Abbreviations: DBP, diastolic blood pressure; MAP, mean arterial pressure; PP, pulse pressure; SBP, systolic blood pressure; Wall‐to‐lumen ratio, intima‐media thickness/carotid diastolic diameter.

**FIGURE 1:**
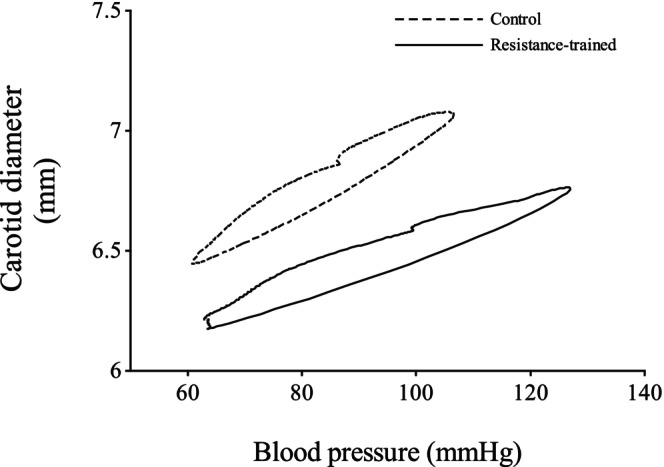
These curves show averaged hysteresis loops of resistance‐trained men (solid line) and age‐matched controls (dotted line). These hysteresis loops are composed of real pressure and vessel diameter.

**FIGURE 2:**
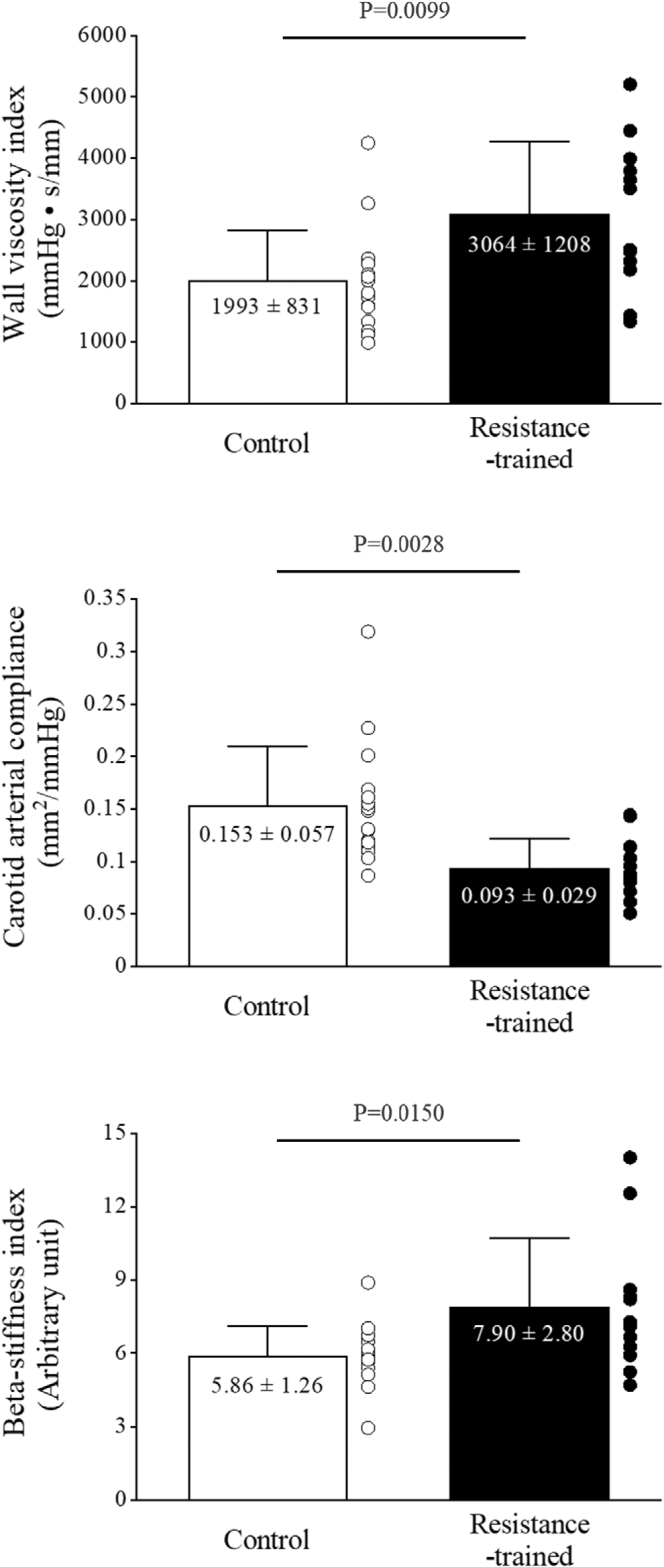
Bar graph showing wall viscosity index (top), dynamic carotid arterial compliance (middle), and beta‐stiffness index (bottom) in control men (white) and resistance‐trained men (black). Jitter plots in each bar show individual data points. Data are means ± SD.

**FIGURE 3:**
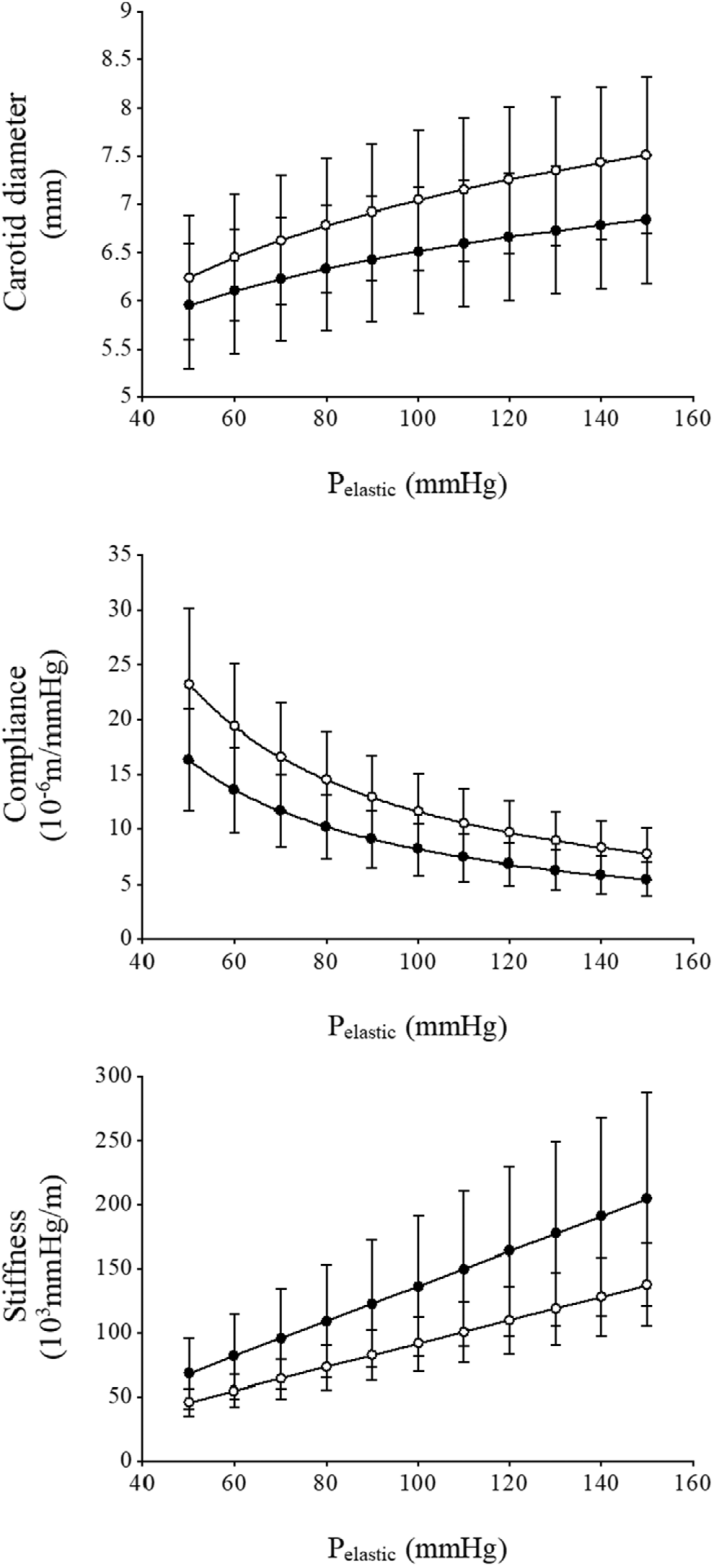
Line graphs showing diameter‐pressure (top), compliance‐pressure (middle), and stiffness‐pressure (bottom) curves over a pressure range from 50 to 150 mmHg in age‐matched control men (white) and resistance‐trained men (black). These graphs were calculated using the values of alpha and beta in Table 3. Data are means ± SD.

Figure 1 shows hysteresis loops for both resistance‐trained and control men. These loops were used to calculate dynamic arterial compliance, beta‐stiffness index, and wall viscosity index. Table 3 shows the arterial elastic properties. The dynamic arterial compliance (0.093 ± 0.029 vs. 0.153 ± 0.057 mm^2^/mmHg: *p* = 0.003, effect size: 0.54) and distensibility (1.55 ± 0.53 vs. 2.18 ± 0.76 10^−3^ mm/mmHg: *p* = 0.021, effect size: 0.44) were lower in resistance‐trained men than in control men, but the wall viscosity index (3064 ± 1208 vs. 1993 ± 831 mmHg·s/mm: *p* = 0.010, effect size: 0.48) and beta‐stiffness index (7.90 ± 2.80 vs. 5.86 ± 1.26 AU: *p* = 0.015, effect size: 0.45) in the carotid artery were significantly higher in resistance‐trained men compared with control men (Figure 2). Figure 3 illustrates the diameter‐pressure, compliance‐pressure, and stiffness‐pressure relationships for both groups. The diameter‐pressure and compliance‐pressure curves for the resistance‐trained men were shifted toward lower values, while the stiffness‐pressure relationship was shifted toward higher values compared to those in the control men. Indeed, the alpha values of Equation 2 were higher in the resistance‐trained men compared with those of control men, while the beta values were lower. To verify these results, we calculated static compliance and stiffness at each subject's effective mean arterial pressure and at a standard isobaric pressure (83.4 mmHg, which is the average mean arterial pressure among all subjects). As a result, effective and isobaric compliance were lower in resistance‐trained men than control men, while effective and isobaric stiffness values were higher. The relationships between arterial wall viscosity and either intima‐media thickness or wall‐to‐lumen ratio were not statistically significant.

**TABLE 3:** Arterial viscoelastic properties.

	Control	Resistance‐trained	*p* Value
Wall viscosity index, mmHg･s/mm^2^	1993 ± 831	3064 ± 1208	0.010
Arterial compliance, mm^2^/mmHg	0.153 ± 0.057	0.093 ± 0.029	0.003
Beta stiffness index, AU	5.86 ± 1.26	7.90 ± 2.80	0.015
Distensibility, 10^−3^ mm/mmHg	2.18 ± 0.76	1.55 ± 0.53	0.021
Alpha value, mmHg/mm	1.683 ± 1.397	2.751 ± 1.193	0.043
Beta value, mm	1.164 ± 0.347	0.817 ± 0.235	0.006
Effective compliance, 10^−6^ m/mmHg	14.79 ± 5.02	9.24 ± 2.78	0.002
Isobaric compliance, 10^−6^ m/mmHg	13.94 ± 4.16	9.79 ± 2.82	0.006
Effective stiffness, 10^3^ mmHg/m	72.7 ± 17.5	118.8 ± 41.1	<0.001
Isobaric stiffness, 10^3^ mmHg/m	76.2 ± 18.0	113.7 ± 46.1	0.006

*Note*: Data are means ± SD. Alpha and beta values were defined each constant of equation “*D* = *α* + *β*・ln *P*_elastic_”. Effective compliance and stiffness were determined at mean arterial pressure in each subject. Isobaric compliance and stiffness were determined at pressure equal to 83.4 mmHg (average value of mean arterial pressure in all subjects).

Table 4 shows cardiac indices in the two groups. Although there was no significant difference in stroke volume between the two groups, fractional shortening and ejection fraction were lower in the resistance‐trained men compared with the control men. Relative wall thickness and left ventricular mass were higher in the resistance‐trained men than in the control men, but the difference in left ventricular mass disappeared when corrected for body surface area.

**TABLE 4:** Cardiac indices.

	Control	Resistance‐trained	*p* Value
Stroke volume, mL	63.8 ± 12.5	72.6 ± 12.1	0.072
Fractional shortening, %	33.9 ± 3.4	28.3 ± 4.7	**0.001**
Ejection fraction, %	62.6 ± 4.7	54.0 ± 7.2	**<0.001**
Relative wall thickness	0.43 ± 0.07	0.71 ± 0.21	**<0.001**
Left venticular mass, g	173 ± 38	229 ± 48	**0.002**
Left venticular mass index, g/cm^2^	96.8 ± 20.6	110.3 ± 20.5	0.097

*Note*: Data are means ± SD. *p* values indicating statistical significance (*p* < 0.05) are shown in bold.

## 4 DISCUSSION

The main findings of the present study are as follows. First, the carotid arterial wall viscosity index was significantly higher in resistance‐trained young men than in sedentary control young men. Second, the resistance‐trained men exhibited lower dynamic arterial compliance and higher beta‐stiffness index compared with sedentary peers. Third, left‐ventricular concentric hypertrophy was observed in resistance‐trained men. To our knowledge, this is the first study to identify central arterial wall viscosity in resistance‐trained young men.

In the present study, resistance‐trained men exhibited higher arterial stiffness and lower arterial compliance compared with age‐matched sedentary control men, consistent with findings from previous studies (Kawano et al., 2008; Miyachi et al., 2003). Moreover, we demonstrated that the carotid arterial wall viscosity index was significantly greater in resistance‐trained men than in sedentary control men. These results suggest that the higher arterial wall viscosity in resistance‐trained men may be a compensatory adaptation for the reduced arterial compliance and increased arterial stiffness induced by resistance training. Arterial stiffening associated with resistance training has been identified in both cross‐sectional (Kawano et al., 2006, 2008; Miyachi et al., 2003) and longitudinal studies (Miyachi et al., 2004). During each bout of high‐intensity resistance exercise, arterial blood pressure is known to increase to levels as high as >320/250 mmHg (MacDougall et al., 1985). To counter this intermittent elevation in blood pressure during resistance exercise, it is generally believed that arterial structures adapt by reducing arterial compliance. However, although pulsatile energy from the heart is converted to elastic energy in the elastic arteries, it remains unclear what happens to the remaining energy when arterial compliance, which represents this elastic energy, is reduced by resistance training. The present study was the first to determine where the remaining pulsatile energy from the heart that was not converted into elastic energy is directed. The results of the present study suggest that resistance‐trained men dissipate a greater proportion of pulsating energy as viscous rather than elastic energy, highlighting a unique adaptation to resistance training.

Arterial wall viscosity is primarily influenced by vascular smooth muscle, as confirmed by Armentano et al., who indicated that wall viscosity was substantially attenuated by approximately 50% under conditions of vascular smooth muscle null tonus (sympathetic nerve inactivation) (Armentano et al., 2006). This was demonstrated using carotid arteries from brain‐dead humans in vitro. Intense resistance training has been shown to be a strong stimulus for increasing sympathetic nervous system activity (Pratley et al., 1994; Raastad et al., 2001), which may contribute to increased arterial stiffness. This effect is likely mediated by chronic restraint on the arterial wall through enhanced sympathetic adrenergic vasoconstrictor tone (Failla et al., 1999). Given these findings, it is plausible to hypothesize that the observed higher arterial wall viscosity in resistance‐trained men is influenced by sympatho‐excitation induced by habitual resistance training.

The previous study indicated that carotid wall viscosity is related to intima‐media thickening in individuals with hypertension (Armentano et al., 1998). However, in the present study, resistance‐trained men with greater arterial wall viscosity did not show higher intima‐media thickness. Moreover, there was no association between arterial wall viscosity and intima‐media thickness in the entire pooled population. Although there were no significant differences in carotid diameter or intima‐media thickness between the two groups, the observed trend toward a higher wall‐to‐lumen ratio (intima‐media thickness/carotid diastolic diameter) in resistance‐trained men suggests that underlying structural changes cannot be completely ruled out. It is widely accepted that the elastic component is attributed to elastic fibers, whereas the viscous component is primarily associated with smooth muscle cells (Wells et al., 1999) or collagen fibers and proteoglycans (Wang et al., 2013). In individuals with hypertension, it is found lower arterial compliance as well as higher arteria wall viscosity (Armentano et al., 2006), which can be attributed to altered arterial and blood flow pressures due to increases in wall thickening, collagen deposition, or elastin fragmentation (Zieman et al., 2005). In addition, considering that aerobic training not only increases arterial extensibility but also enhances elastin content in the vessel while decreasing the calcium content within elastin (Matsuda et al., 1993), it cannot be ruled out that resistance training affects vascular structure, potentially increasing viscosity and reducing compliance in elastic arteries.

Exertional hypertension induced by acute bouts of resistance exercise has been shown to reduce endothelial function, as assessed by endothelium‐dependent flow‐mediated dilation (Morishima et al., 2018). Additionally, lower‐extremity weightlifting exercise acutely impairs brachial endothelial function in sedentary individuals but not in trained weightlifters (Jurva et al., 2006). Regarding the relationship between long‐term resistance training and endothelial function, several studies have reported that a few months of intense resistance training do not significantly affect endothelial function (Kawano et al., 2008; Rakobowchuk et al., 2005). Therefore, given the current evidence, no significant decrease in endothelial function has been observed with resistance training, suggesting that endothelial dysfunction is unlikely to contribute to the increased arterial wall viscosity associated with resistance training. However, considering the transient adverse effects of a single bout of resistance exercise on endothelial function, this possibility cannot be completely ruled out.

The heart undergoes morphological adaptations in response to long‐term training, with resistance training and aerobic training inducing concentric and eccentric left ventricular hypertrophy, respectively. In the present study, resistance‐trained men exhibited a trend toward left ventricular hypertrophy compared with sedentary control men; however, there was no significant difference in stroke volume between the two groups. These results indicate that habitual resistance training induces concentric left ventricular hypertrophy but does not alter the ejection of cardiac pulsatile energy into the arteries. Therefore, these findings suggest that, in resistance‐trained men, concentric hypertrophy may be associated with reduced arterial compliance or increased arterial wall viscosity.

In the present study, both isobaric and effective compliance were significantly lower in resistance‐trained men compared to sedentary controls. In interpreting these compliance data, it is important to distinguish between isobaric and effective compliance. Isobaric compliance is calculated at a standardized pressure level, enabling comparison of arterial mechanical properties across participants under uniform conditions. Effective compliance, by contrast, is determined at each individual's actual mean arterial pressure and thus represents a physiologically relevant condition. While both indices offer meaningful insights, the exclusion of participants with excessively high blood pressure in our sample minimized the differences between the two measures. Nonetheless, care must be taken in interpreting these parameters, particularly when comparing populations with broader blood pressure variability. Furthermore, to better characterize arterial behavior under dynamic loading, we analyzed carotid arterial distensibility. This parameter, which is less influenced by vascular geometry and pressure normalization, was significantly lower in resistance‐trained men than in controls. This finding supports the notion that resistance training alters the intrinsic viscoelastic properties of the arterial wall beyond simple changes in arterial structure or loading conditions.

We previously reported the greater arterial wall viscosity in endurance‐trained athletes compared with age‐matched control men (Kawano et al., 2021). The result and the present findings are consistent in showing that both endurance‐ and resistance‐trained men exhibit higher arterial wall viscosity compared to healthy young men in the general population. The higher arterial wall viscosities observed in endurance‐trained men and resistance‐trained men are quite similar in the sense that they result from the dissipation of pulsatile energy, which should have been converted into elastic energy. However, in endurance‐trained individuals, particularly young people, the increased pulsatile energy from the heart, instead of being converted into elastic energy, is dissipated as viscosity because the arterial compliance has reached its upper limit. On the other hand, the increase in arterial wall viscosity induced by resistance training reduces arterial compliance, which may differ from the adaptation of arterial elasticity to endurance training.

Higher arterial wall viscosity and lower compliance of the central artery in resistance‐trained men were similar to those observed in individuals with hypertension (Armentano et al., 2006). Additionally, the previous study has reported an increase in arterial wall viscosity along with arterial stiffening with aging (Kawano et al., 2021). Given these findings, the increase in arterial wall viscosity due to hypertension or aging may represent a compensatory adaptation to enhanced vascular stress. Thus, while increased arterial wall viscosity suggests an elevated risk of cardiovascular disease—similar to that seen in atherosclerosis due to aging or hypertension—it may, in fact, serve as a reasonable physiological adaptation. Indeed, a previous systematic review reported that a longer duration of muscle‐strengthening activities was associated with a higher risk of cardiovascular disease mortality (Momma et al., 2022). On the other hand, the higher arterial wall viscosity and lower arterial compliance in resistance‐trained men may represent a physiological adaptation to prevent vessel damage and rupture caused by the high pressor response during high‐intensity resistance exercise. Future intervention studies on resistance training in various populations, along with detailed investigations into the mechanisms underlying arterial viscoelastic adaptation, will help elucidate the clinical significance of increased arterial stiffness and arterial wall viscosity in response to resistance training.

The present study had some limitations. First, the resistance‐trained men who participated in this study had been training for more than 4 years; however, the duration varied among individuals. This means that while the presence or absence of training was clearly defined, we were unable to account for the potential effects of the duration of arterial exposure to training. Second, because the present study had a cross‐sectional design, we could not determine the underlying mechanisms of increased arterial wall viscosity in response to resistance training. Third, the participants in this study were exclusively young males. Therefore, future studies should include middle‐aged and older adults as well as women.

## 5 CONCLUSION

The present study indicated that central arterial wall viscosity is higher and arterial compliance is lower in resistance‐trained men than in age‐matched sedentary men. These findings may suggest that resistance‐trained men dissipate a greater proportion of pulsatile energy as viscosity rather than elasticity compared to sedentary men. To further clarify the association between exercise training and arterial wall viscosity, additional studies are necessary.

## AUTHOR CONTRIBUTIONS

HK and MM (Miaychi) conceived and designed the study. HK, NN, KT and MK conducted and performed the experiments. HK and KY analyzed the data. HK prepared the figures and tables. HK, MM (Miyashita) and MM (Miyachi) drafted the manuscript. SS and MM (Miyachi) supervised the study and provided critical revisions; all authors reviewed and approved the final version.

## FUNDING INFORMATION

This Study was supported by Grants‐in‐Aid for Scientific Research 18K10826 (to Kawano H) from the Japan Society for the Promotion of Science.

## CONFLICT OF INTEREST STATEMENT

None for all authors.

## Data Availability

The data supporting the findings of this study are available from the corresponding author upon reasonable request.
